# Prediction and prevention of late-onset pre-eclampsia: a systematic review

**DOI:** 10.3389/fmed.2024.1459289

**Published:** 2024-11-21

**Authors:** Anna Baylis, Wei Zhou, Ellen Menkhorst, Evdokia Dimitriadis

**Affiliations:** ^1^Department of Obstetrics, Gynaecology and Newborn Health, University of Melbourne, Parkville, VIC, Australia; ^2^Gynaecology Research Centre, Royal Women's Hospital, Parkville, VIC, Australia

**Keywords:** late-onset pre-eclampsia, prediction, prevention, risk factor, management

## Abstract

**Background:**

Pre-eclampsia is a major cause of perinatal morbidity and mortality worldwide. Late-onset pre-eclampsia (LOP), which results in delivery ≥34 weeks gestation, is the most common type. However, there is a lack of knowledge in its prediction and prevention. Improving our understanding in this area will allow us to have better surveillance of high-risk patients and thus improve clinical outcomes.

**Methods:**

A systematic review was performed using a search of articles on PubMed. The search terms were ((late-onset) AND (pre-eclampsia)) AND ((risk factor) OR (risk) OR (prediction) OR (management) OR (prevention)). Primary literature published between 1 January 2013 and 31 December 2023 was included. Human studies assessing the prediction or prevention of late-onset pre-eclampsia were eligible for inclusion.

**Results:**

Sixteen articles were included in the final review. The key risk factors identified were Body Mass Index (BMI), chronic hypertension, elevated mean arterial pressures (MAPs), nulliparity, and maternal age. No clinically useful predictive model for LOP was found. Initiating low dose aspirin before 17 weeks gestation in high-risk patients may help reduce the risk of LOP.

**Conclusion:**

While aspirin is a promising preventor of LOP, preventative measures for women not deemed to be at high-risk or measures that can be implemented at a later gestation are required. Biomarkers for LOP need to be identified, and examining large cohorts during the second or third trimester may yield useful results, as this is when the pathogenesis is hypothesized to occur. Biomarkers that identify high-risk LOP patients may also help find preventative measures.

## 1 Introduction

Pre-eclampsia is a multi-system disorder of pregnancy, defined as new-onset hypertension (>140/90 mmHg), after 20 weeks gestation, with evidence of maternal systemic involvement such as proteinuria, liver transaminitis, neurological dysfunction, and hematological changes. It affects approximately 4.6% of pregnancies (1) and is associated with 10–15% of maternal deaths worldwide (2). Preeclampsia is generally classified as early-onset (EOP, delivery at <34 weeks gestation) and late-onset (LOP, delivery at ≥34 weeks gestation) (3, 4). It may present with headaches, abdominal pain, foetal growth restriction and oedema, or less commonly with visual disturbances, seizures and oliguria (5, 6).

Current research suggests that EOP and LOP have different aetiologies (3, 7). EOP likely arises from altered decidual spiral artery remodeling during placentation, leading to deficient blood flow to the placenta, placental hypoxia, and syncytiotrophoblast dysfunction, which causes disturbed production of angiogenic and pro-inflammatory factors (8, 9). EOP is also often associated with fetal growth restriction (7). Like EOP, LOP is associated with syncytiotrophoblast dysfunction, causing disturbed production of angiogenic and pro-inflammatory factors, but this occurs later in pregnancy (9, 10). Further in LOP there is no histopathological evidence for altered decidual spiral artery remodeling during placentation (9, 10). An imbalance of angiogenic factors/anti-angiogenic factors, particularly low levels of PlGF, may contribute to hypo-perfused placental lesions in LOP (9). As gestation increases, synciotrophoblast stress increases as well as endothelial cell dysfunction (11, 12). This has led to thoughts about pre-existing maternal conditions such as obesity, hypertension and diabetes contributing to LOP (11). The different pathologies for EOP and LOP may explain why biomarkers used for the prediction of EOP are not effective for LOP.

While there are predictive biomarkers and a preventative treatment for EOP, there remains a significant knowledge gap in the prediction and prevention of LOP. This is concerning as LOP is seven times more common than EOP (13) and is associated with severe birth outcomes, perinatal death, and cardiovascular disease (13, 14). Having predictors in place for LOP will allow for better surveillance of these patients, and improved clinical outcomes. There is currently no screening tool or preventative measures for LOP specifically.

To the best of our knowledge, there are no systematic reviews summarizing the current literature on the prediction and prevention of LOP. This systematic review evaluates primary literature on LOP published since 2013. It aims to enhance understanding of the risk factors, predictive models, and prevention strategies for LOP.

## 2 Materials and methods

### 2.1 Search strategy

This systematic review was conducted through a search of articles on PubMed published on or before 31 December 2023. The key words used were ((late-onset) AND (pre-eclampsia)) AND ((risk factor) OR (risk) OR (prediction) OR (management) OR (prevention)). The search was limited to articles published from 2013 onwards, including case reports, clinical studies, comparative studies, evaluation studies, multicentre studies, observational studies, and randomized control trials.

### 2.2 Inclusion criteria

Human studies assessing the prediction or prevention of LOP were eligible for inclusion. Articles that examined both LOP and EOP were included if these phenotypes were divided in the study’s results.

### 2.3 Exclusion criteria

Exclusion criteria included manuscripts which did not investigate LOP prediction or prevention, did not define LOP as delivery >34 weeks, lacked a specific focus on LOP, were not available in English, or were based on animal models. Narrative reviews, systematic reviews, meta-analysis and validation studies were also excluded.

### 2.4 Article selection

Articles identified in the search were reviewed by two independent authors (AB and WZ). If there was disagreement, a third reviewer (ED) was consulted. Articles that did not meet the inclusion criteria were identified by reading through the titles and abstracts and subsequently removed. The remaining articles were carefully read-through, and those not meeting the inclusion criteria were removed.

## 3 Results

**Figure 1:**
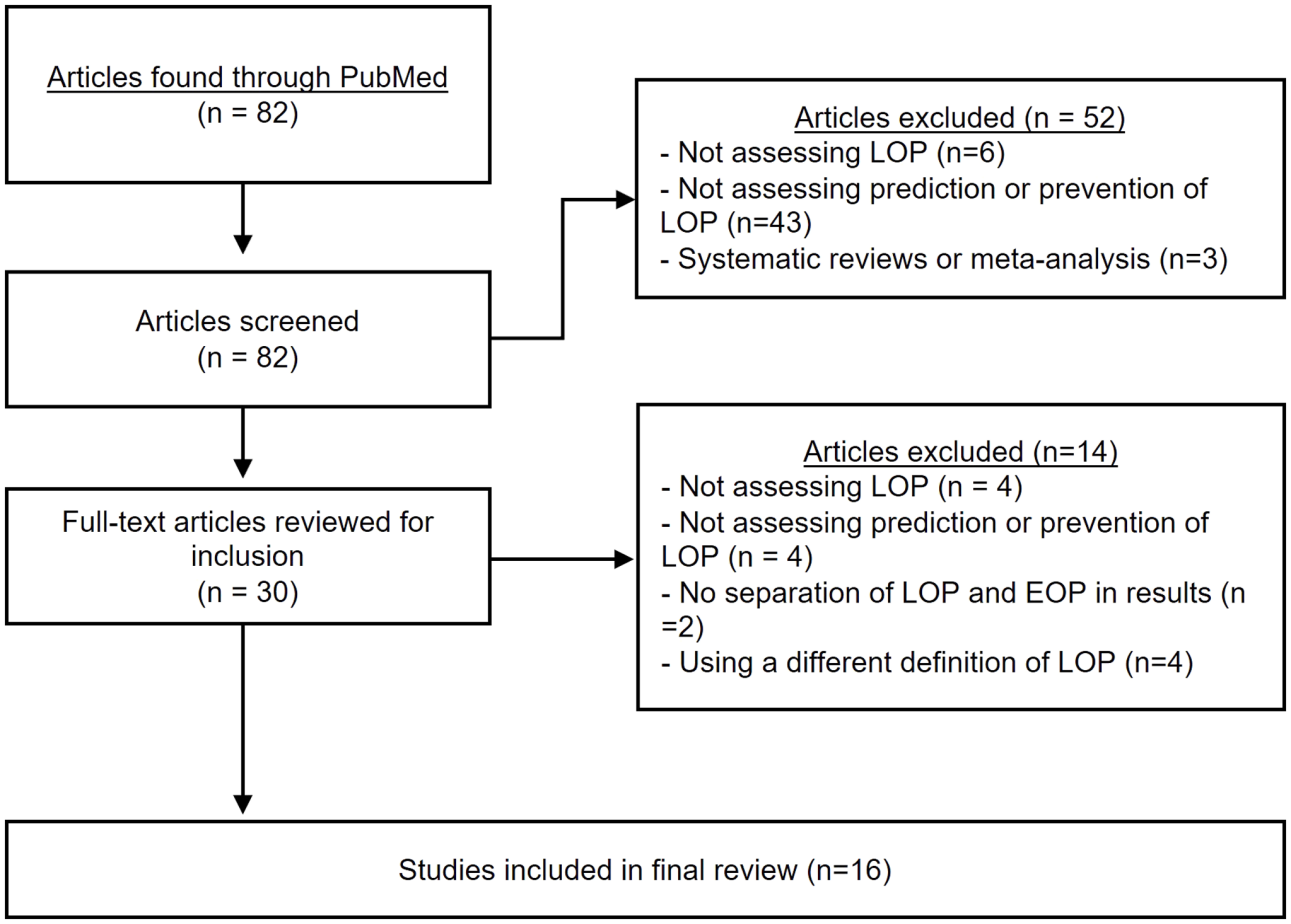
PRISMA flowchart. The above chart shows the different steps taken during the systematic review process.

A total of 82 articles were identified from the search strategy (Figure 1), but 52 were excluded during the title and abstract screening. Subsequently, 30 full-text articles were reviewed, with 14 further removed based on the criteria listed in Figure 1. Thus, a total of 16 articles were ultimately included in the review.

### 3.1 Risk factors

The results of the studies included that looked at prediction and risk factors of LOP are summarized in Table 1. They covered a broad range of predictors, including maternal characteristics, serum biomarkers and small nucleotide polymorphisms.

**Table 1:** Summary of studies on prediction and risk factors of late-onset pre-eclampsia.

Author, year	Sample size	Study type	Relevant results
Robillard et al., 2019 (15)	LOP *n* = 1,162 Controls *n* = 71,078	18-year retrospective observational study	Incremental increases in BMI are associated with increasing incidence of LOP. Significant risk factors identified for LOP were advanced age OR 1.03 [1.02–1.04], chronic hypertension OR 4.95 [3.6–6.3], BMI OR 1.05 [1.04–1.06] and nulliparity OR 2.44 [2.1–2.8]. Chronic hypertension was the greatest independent risk factor for LOP *p* < 0.001.
Bendix et al., 2023 (16)	LOP *n* = 27 Controls *n* = 194	Case control study	No significant differences in first-trimester serum alipoprotein levels were found between the LOP and control group. The best performing screening model combined parity, age, BMI, MAP, ApoD, ApoB-100 and gave an AUC value of 0.87, sensitivity of 55.5% with 95% CI [30.3;80.7] for a false positive rate of 10%. Women with LOP had higher BMIs, MAPs and were mostly nulliparous (*p* < 0.05).
Iacobelli et al., 2017 (18)	LOP *n* = 933 Controls *n* = 59,665	Retrospective observational cohort study	There was a significant association between LOP and pre-existing diabetes, GDM, BMI > 30, IVF, kidney disease, hypercholesterolaemia (*p* < 0.05).
Murtoniemi et al., 2018 (20)	Total *n* = 257 LOP *n* = 34	Prospective cohort study	Lower levels of first-trimester HCG-h and higher MAPs were associated with LOP. The model with the highest prediction rates used the variables: age, prior PE, prior SGA DM-type 1, MAP, prior fetus mortus, hCG, %hCG-h, free beta hCG, PIGF, Uta-PI with an AUC value 0.66 with 32% sensitivity at 90% specificity.
Nguefact et al., 2018 (17)	LOP *n* = 112 EOP *n* = 58	5-month prospective cross-sectional study	LOP was significantly associated with new paternity, and nulliparity (*p* < 0.05).
Sanhal et al., 2016 (23)	LOP *n* = 36 Controls *n* = 31	Comparative study	Plasma fetuin A levels were significantly higher in the LOP group compared to controls p < 0.001, measured at gestation >34 weeks. Plasma fetuin A levels gave an AUC of 0.196, 95% CI [0.085,0.306]
He et al., 2016 (24)	LOP *n*-30 Controls *n* = 30	Case–control study	Serum levels of complement factors C1q, Bb, C3a, C5a and MAC measured >34 weeks gestation, were significantly higher in LOP compared to control group *p* < 0.05. The LOP group was significantly associated with increased age and BMI (*p* < 0.05)
Uiterweer et al., 2020 (21)	Pittsburgh population: LOP *n* = 33 Controls *n* = 25 Dutch population: LOP *n* = 95 Controls *n* = 469	Case–control study	Women with LOP were significantly associated with having lower relaxin levels at 9–13 weeks gestation, below the 10th centile in the Pittsburgh group OR 5.29 [1.1–25.5] and the Dutch group OR 2,03 [1.06–3.88]. Relaxin levels improved the detection rate of LOP by 2.5% in a prediction model combining maternal characteristics (age, BMI, nulliparity) and MAP. Women who developed LOP were also associated with a significantly higher first-trimester MAP, BMI and nulliparity (*p* < 0.05).
Sonek et al., 2018 (25)	LOP *n* = 33 Control *n* = 1,022	Prospective observational cohort study	Frist trimester screening of LOP using maternal characteristics (ethnicity, chronic hypertension, smoking status, parity, family history, BMI) had a low detection rate of 15% for 5% false positive. This was not improved by the addition of biomarkers or placental characteristics. The only biomarker statistically significant in LOP compared to the control group was MAP *p* < 0.001.
Verlohren et al., 2014 (26)	LOP *n* = 1802 Total *n* = 26,893	Retrospective observational cohort study.	No association was found between uterine artery resistance index and LOP (*p* > 0.05). There was a high prevalence of SGA and LGA neonates for the LOP group.
Burgess et al., 2019 (19)	LOP *n* = 126 Controls *n* = 259	Retrospective observational study	After controlling for GDM, CHTN, and DM, LOP was no longer significantly associated with having blood type AB OR 2.53 95% CI [0.7–9.2].
Andersen et al., 2019 (27)	Total *n* = 501	Retrospective observational study	The predictive performance of sFlt-PIGF ratio with a threshold of 66 was AUC 0.85 with 95% CI [0.8–0.9] within 4 weeks of developing LOP.
Altemani et al., 2021 (22)	LOP *n* = 11 Matched controls *n* = 22 Total controls *n* = 202	Case–control study	Obese LOP women had significantly lower levels of butyrate-producing gut bacteria and serum butyrate at 28 weeks gestation compared to age, parity and BMI-matched controls. The LOP group had significantly higher serum triglyceride and VLDL levels (*p* < 0.05).
Niu et al., 2017 (28)	LOP *n* = 584 Controls *n* = 1,263	Case–control study	Significant differences were found in distributions for SNP IL-22 rs2227485 between the LOP and control group *p* = 0.002, OR 1.125 [0.977–1.295]. Significant differences were found between LOP and controls in genotypic and allelic frequencies of SNP IL-22RA1 rs3795299 *p* < 0.001, OR 1.355 [1.165–1.576].

LOP, late-onset pre-eclampsia; PE, pre-eclampsia; MAC, membrane attack complex; MAP, mean arterial pressure; HCG-h, hyperglycosylated human chorionic gonadotrophin; AUC, area under the receiver operating characteristic curve; CI, confidence interval; SGA, small for gestational age; LGA, large for gestational age; GDM, gestational diabetes mellitus; CHTN, chronic hypertension, DM, diabetes mellitus; sFlt, soluble fms-like tyrosine kinase; PIGF, placental growth factor; BMI, body mass index; VLDL, very low-density lipoprotein; SNP, small nucleotide polymorphism; IL, interleukin.

Maternal characteristics such as BMI, age, nulliparity and hypertension were identified as risk factors for LOP. Incremental increases in BMI had a positive linear correlation with LOP in an observational study in Reunion Island among a cohort of 72,920 women (15). Obesity and LOP rates both increased by 11 and 12%, respectively, over the 18-year period (15). Among the same cohort, the significant risk factors for LOP were chronic hypertension, increasing BMI, nulliparity and increasing maternal age (15). In agreement, a recent study from Denmark found that women who developed LOP were mostly nulliparous and had significantly higher BMIs and blood pressure (16). A questionnaire survey of 112 women with LOP in Doula, Cameroon, found that LOP was associated with new paternity and nulliparity (17). Another cohort study in Reunion Island found that IVF, renal disease, gestational diabetes mellitus (GDM) and hypercholesterolaemia were significantly associated with LOP (18). Maternal ABO blood type has been investigated for its association with LOP (19). After excluding for individuals with gestational diabetes, chronic hypertension, and diabetes, the association between AB blood type and LOP development was not significant (19).

### 3.2 Biomarkers

#### 3.2.1 Prediction

A range of serum biomarkers were evaluated for their role in predicting LOP. One study found an association between lower percentages of hyper-glycosylated human chorionic gonadotrophin (HCG-h) in the first trimester and the development of LOP (20). The addition of the biomarkers HCG-h, free b-HCG, PIGF and UtA-PI MoM, improved the sensitivity of their predictive model from 10 to 32% (20). First trimester serum apolipoproteins were not found to be significantly different between LOP and controls (16). Women with LOP were significantly associated with low first-trimester relaxin hormone levels, below the 10th centile (21).

Another study investigated the levels of butyrate-producing bacteria in the gut of women with LOP and obesity at 28 weeks gestation (22). They demonstrated that these women with LOP had significantly lower levels of butyrate-producing bacteria in their gut and lower serum butyrate compared to BMI-matched controls who did not go on to develop LOP (22). Low levels of butyrate in the LOP group was also associated with significantly higher serum triglyceride and VLDL levels at 28 weeks gestation compared to controls (22).

#### 3.2.2 Diagnosis

Plasma fetuin A (FA) levels were significantly higher in 36 patients with LOP compared to 31 gestational-age-matched controls (23). However, plasma FA levels were not useful in discriminating between LOP and controls (23). Another study found complement factor levels, specifically C1q, Bb, C3a, C5a and MAC were significantly elevated in serum in LOP compared to controls even after correcting for BMI (24).

### 3.3 Placental characteristics

A number of studies investigated placental characteristics in predicting LOP. One prospective observational cohort study used a combination of maternal characteristics (ethnicity, chronic hypertension, smoking status, parity, family history, BMI), biomarkers (PIGF, PAPP-A, MAP, MSAFP, UtA-PI), and estimated placental volumes to determine if a first-trimester screening tool for pre-eclampsia could be developed (25). The first trimester screening performance of LOP was low, with detection rates of 15 and 48% for 5 and 10% false positive rates, respectively, whereas the detection rate for EOP was 85% (25). Elevated or reduced uterine artery resistance index at 18 to 23 + 6 weeks gestation was not associated with LOP in a retrospective observational study (26). However, LOP patients had a high prevalence of small for gestation age (SGA) and large for gestational age (LGA) births (26). LGA neonates born to LOP patients, were not associated with a low uterine artery mean resistance index (26). Using the sFlt-1/PIGF serum ratio to predict the onset of pre-eclampsia was found to be less accurate for LOP than EOP (27). The optimal ratio threshold for predicting both LOP and EOP within 1 to 4 weeks was found to be 66, and this had a high negative predictive value for LOP of 86–93% (27).

### 3.4 Single nucleotide polymorphisms

Single nucleotide polymorphisms (SNP)s have been investigated in mothers in association with pre-eclampsia. SNPs in IL-22 and IL-22 receptor alpha 1 (IL-22RA1) have been found to be associated with LOP in Chinese Han women (28). Significant differences in the distributions have been found for the SNP IL-22 rs2227485 between LOP women and controls (28). There were also significant differences for genotypic and allelic frequencies of the SNP IL-22RA1 RS3795299 between the LOP and controls (28).

### 3.5 Aspirin for prevention

The results of the studies on prevention of LOP are summarized in Table 2. Initiation of low-dose aspirin prior to 17 weeks gestation in high-risk pregnant women has been found to be protective against the development of LOP in a secondary analysis of a randomized control trial (29). The rate of LOP was significantly reduced in the low-dose aspirin group versus the placebo: 17.36% vs. 24.42% with *p* = 0.047 (29). There was also a significant reduction of LOP in women with chronic hypertension who took low-dose aspirin (29). Another study found that proper adherence to aspirin leads to a decreased incidence of LOP in women with pre-existing DM, chronic hypertension, systemic lupus erythematosus, or a history of pre-eclampsia (30). It was found that 44% of women had inadequate adherence to taking aspirin (30). Within this low-adherence group, 41% developed LOP, while only 5% developed LOP in the adequate-adherence group (30).

**Table 2:** Summary of studies on prevention of late-onset pre-eclampsia.

Author, year	Sample size	Study type	Relevant results
Moore et al., 2015 (29)	Total *n* = 523 Aspirin group *n* = 265 Placebo *n* = 258	Randomised control trial: secondary analysis	Daily aspirin given to high-risk pregnant women significantly reduced the rate of LOP compared to the placebo with *p* = 0.047.
Shanmugalingham et al., 2020 (30)	Total *n* = 187	Prospective observational cohort study	44% of high-risk women had inadequate adherence to aspirin. Women <90% adherent had significantly higher incidence of LOP with OR 4.2, 95% CI [1.4, 19.8]. Adequate adherence to aspirin reduced the incidence of LOP with *p* < 0.001.

### 3.6 Quality assessment

The quality assessment of this systematic review reveals risks primarily associated with limitations in the search strategy, study selection, and potential publication bias. Despite efforts to mitigate bias by including articles with diverse findings, inherent publication bias may persist, especially including studies with significant results. Restricting the search to PubMed articles may also overlook relevant studies from other databases. Variation in late-onset pre-eclampsia definitions among included studies could impact result generalizability. Inclusion of studies with small sample sizes and conflicting results may introduce bias, reducing certainty in conclusions. Although attempts were made to reduce bias, study selection and potential publication bias should be considered when interpreting results.

### 3.7 Limitations of present review

This review had several limitations. The search terms identified few recent studies. Inconsistent definitions of LOP among studies led to their exclusion, limiting result scope. Some studies may not have used the term “late-onset,” potentially causing missed articles. Most of the included studies had small sample sizes, likely reducing their statistical power, while conflicting results hampered drawing definitive conclusions. Variations in methods and timing of predictor assessment across studies additionally complicated result comparison.

## 4 Discussion

The present review analysed primary literature on the prediction and prevention of LOP. While several risk factors have been found, there is no clear clinical model for predicting or preventing LOP.

### 4.1 Prediction

The research demonstrates it is difficult to predict LOP in the first trimester. Among the studies included, no first trimester screening model could reliably predict LOP (16, 20, 25). This is consistent with the proposed mechanism, that the pathogenesis of LOP occurs later in pregnancy (31), suggesting that many of the biomarkers may not be observable until the second or third trimester. While first trimester serum apolipoproteins were not significantly different between LOP and controls, they may be useful for first-trimester screening but the study sample size of 27 patients was small (16). Furthermore, this study only assessed serum apolipoproteins once throughout pregnancy, and did not specify the gestation week (16), limiting its utility. It would benefit from a larger sample size and more precise gestational age definitions. Women with LOP were significantly associated with low first trimester serum relaxin levels, which may serve as a promising predictive biomarker (21). However, the first trimester serum relaxin hormone levels improved the detection rate of LOP by only 2.5% when combined with maternal characteristics (age, BMI, and nulliparity) and MAP (21). The sample size of 120 patients was also small (21). It would be beneficial to see if there are any changes in the serum apolipoproteins and relaxin levels for LOP patients later in pregnancy as this is when the pathogenesis of LOP is hypothesized to occur (31). Similarly, two other studies were unable to find a useful first trimester predictive model for LOP (20, 25). However, one study found that LOP patients (*n* = 34) had a significantly lower first trimester percentage of hyperglycosylated (h) HCG than women who did not develop LOP (*n* = 223) (20). This suggests that percentage h-HCG may be able to serve as a predictive biomarker for LOP or play a part in predictive models. However, the study only included women deemed to be at high risk of PE and did not specify how they determined this. These studies are limited with their small sample sizes which reflects a greater problem with prospective studies on LOP; the relatively uncommon condition means it is difficult to gather large sample sizes. A previous study which was used to inform current aspirin guidelines for the prevention of pre-eclampsia determined that a sample size of >1,600 participants would be required to give adequate power to show effects (32). Hence these studies looking at the prediction of LOP should aim to use sample sizes of a similar scale.

Many inflammatory biomarkers are elevated during the manifestation of LOP. Both classical and alternative complement pathways were found to be activated during LOP (24). These findings corroborate the hypothesis that pre-eclampsia is a disease of pathological inflammation (33), as the complement system which activates inflammation in the body, has long been associated with inflammatory diseases (34). Detection of complement factors before LOP needs evaluation to decipher if complement factors can serve as predictive biomarkers. The downregulation of inflammation, including that of the complement system may be helpful in the prevention or treatment of LOP but requires further research. One study reported that plasma FA levels were significantly elevated in women with LOP (23) and indicated inflammation is repressed in these women. However, higher levels of FA are seen in other inflammatory diseases like metabolic syndrome, type 2 diabetes and fatty liver disease (35–38). It is unclear if plasma FA is always high in these patients who develop LOP, or if its upregulation is a part of the pathophysiology of LOP. Further research is necessary to assess the role of plasma FA levels in patients prior to the development of LOP, and to evaluate if it can serve as a predictive biomarker of LOP.

According to the studies included, the only predictors of LOP that would be useful in the first trimester are maternal characteristics. A high BMI, nulliparity, new paternity, advanced maternal age, chronic hypertension and high MAP (15–18, 25) were significantly associated with the development of LOP. This is in line with a meta-analysis (39) which also found systemic lupus erythematosus and chronic kidney disease to be risk factors for pre-eclampsia, noting that they did not look at LOP specifically. However, another study is conflicting as they demonstrated that primiparous women are four-times more likely to have EOP than LOP, but later contend that nulliparous women are at higher risk of LOP (17). Furthermore, they do not have a control group, and miss key predictors in their analysis such as BMI. Overall, the studies that used these maternal characteristics to predict LOP, still had low detection rates, and were thus not applicable clinically (16, 20, 25). These maternal characteristics may be used to identify patients at higher risk but are unable to accurately predict those who will develop LOP. Similarly, women with chronic hypertension are more likely to develop LOP due to the underlying vascular dysfunction, which can exacerbate abnormal placental development. However, a previous study has shown that the correlation between chronic hypertension and pre-eclampsia is more pronounced in EOP than in LOP (15).

The finding from the Reunion Island cohort that increasing BMI is a risk factor for LOP (15) is well supported by other literature which shows that pre-eclampsia is associated with pre-pregnancy BMI (39, 40). The Reunion Island study found that as BMI increased, so did the incidence of LOP but not EOP (15), which is of concern as obesity rates are rising world-wide. Interestingly, when controlling for maternal BMI and age, there was no association found between either LOP or EOP and gestational diabetes (15). This contrasts with another study on Reunion Island which found both GDM and pre-existing diabetes to be risk factors for LOP, however they did not control for maternal BMI and age (18). Altogether, this suggests that pre-pregnancy BMI and maternal age may be confounding factors in studies that identify GDM as a risk factor for pre-eclampsia (41–43). A recent study using the SPRING cohort in Australia found that obese patients who developed LOP had significantly higher serum triglyceride and VLDL levels at 28-weeks gestation compared to BMI matched controls (22). This is supported by the Reunion Island study, which also found that hypercholesterolaemia was associated with LOP (18). Measuring lipoproteins may therefore serve to identify which obese patients are at higher risk of developing LOP. By contrast a Danish study which did not separate between EOP and LOP found no differences in BMI between pre-eclampsia and non-pre-eclampsia women (27). This may be because they did not separate the data between EOP and LOP, as the previous study from Reunion Island found that BMI was more closely correlated with LOP than EOP (15). The Reunion Island cohort study is advantageous as it has a large sample size of >71,000 patients including 1,162 with LOP, and its 18-year duration, which allows for the identification of trends over time (15).

Gut microbiota has been shown to be altered in obese patients with LOP in the SPRING study. Lower butyrate producing gut bacteria and serum butyrate levels were significantly associated with LOP in obese patients (22). This indicates that a deficiency in serum butyrate may contribute to the development of LOP. Certain butyrate-producing gut bacteria have been associated with better glycaemic control (44) and serum butyrate supplementation reduces childhood obesity levels (45). This association in children indicates that butyrate supplementation may reduce the risk or help prevent LOP however this requires further investigation. The SPRING study is limited with a sample size of only 11 LOP patients, all of whom were clinically obese (22). The findings should be further explored, through monitoring of butyrate levels at different gestations throughout pregnancy with a greater sample size including obese and non-obese patients.

Many biomarkers that are predictive for EOP are not useful for LOP. By contrast to EOP (25), there was no association found between LOP and uterine artery resistance index or pulsatility index (16, 25, 26). This is in line with a meta-analysis on the utility of uterine artery doppler for predicting pre-eclampsia which found UtA-PI was a better predictor for EOP (46). Additionally, the sFlt/PIGF ratio was found to be a better predictor for EOP than LOP (27). Yet their results have high negative predictive values of 90 and 86% for EOP and LOP, respectively. These findings support the use of the sFlt/PIGF ratio in clinical practice for ruling out pre-eclampsia in women at high risk or with suspicion for developing pre-eclampsia. However, a key limitation in their study was that they only included women who were suspected of developing pre-eclampsia, some of whom were already symptomatic, which may have skewed the results. These findings align with a previous study that found the sFlt-1/PIGF ratio to be more efficient for predicting EOP (47). The limited clinical utility of these markers demonstrates the continuing challenges of predicting LOP.

The Type I vs. Type II model of pre-eclampsia may explain many of the differences in the prediction between EOP and LOP. This way of characterizing pre-eclampsia is based on the phenotypes, and organ dysfunction from a molecular level (48). Type I pre-eclampsia which is generally early-onset, is associated with significant placental dysfunction and this has a greater imbalance of sflt1 and PlGF (48). This may explain why placental characteristics like UTA-PI and sflt/PlGF ratio are better predictors for EOP (16, 25–27) as Type I pre-eclampsia is instigated by placental pathology (48). In contrast Type II pre-eclampsia which is generally late-onset, is suggested to arise from maternal maladaptation to pregnancy arising from the cardiovascular system from underlying endothelial damage (48). This aligns with the studies finding that BMI, hypercholesterolaemia and chronic hypertension are better predictors of LOP (15, 18, 22) as these risk factors may predispose patients to endothelial damage. Overall, this Type I and Type II pre-eclampsia model may be used to form distinct predictive methods for EOP and LOP, based on the pathophysiologies and using this to guide interventions. For example, aspirin administration and placental monitoring may be crucial in preventing and managing Type I. In contrast, lifestyle changes, cardiovascular health management, and vigilant prenatal care are more relevant for Type II.

Heritable markers like blood type and SNPs have been associated with LOP. Significant differences in the SNPs IL-22 rs2227485 and IL-22RA1 rs3795299 between the LOP and controls have been demonstrated (28). However, they do not go into any further detail about the nature of these differences, which makes their findings difficult to interpret. Additionally, their study focuses on the Chinese Han population and further studies could be done to evaluate if these differences are also relevant to other groups. Various studies have found other SNPs to be associated with pre-eclampsia (49–51), but little progress has been made in using these SNPs for predicting LOP. Another study contends that interactions between SNPs and environmental factors will form the genetic basis of pre-eclampsia (52). There is still a long way to go in determining the function of these SNPs and whether they can be used to help predict this disease. Interestingly, while the blood type AB was also associated with LOP (19), after controlling for GDM, chronic hypertension and DM there was no association. Another similar sized study (*n* = 185) found no significant association between any ABO blood type and LOP (53) suggesting blood type is unlikely to predict LOP. It would be beneficial to confirm this by repeating these studies with larger cohorts.

### 4.2 Prevention

Aspirin may be useful in the prevention of LOP. Initiating low-dose aspirin in high-risk women before 17 weeks gestation significantly reduced the rate of LOP (29). Interestingly this study found no significant reduction in EOP (29). Other studies and meta-analyses (32, 54–56) that demonstrate the benefit of aspirin to prevent pre-eclampsia either do not separate pre-eclampsia into subtypes or define it as preterm (<37 weeks) vs. term (≥37 weeks). In particular, prior research demonstrates that aspirin is more effective in preventing preterm pre-eclampsia than term pre-eclampsia (32). Furthermore, this study (29) found that women with chronic hypertension benefitted the most from low-dose aspirin also contradicting the literature (57). Due to the contrasting results, it may be necessary to repeat their study with a larger sample size, and in a prospective nature to ensure adequate control and validity. However, adherence to aspirin in pregnancy is proven problematic with one study finding only 56% of women adherent to the prescribed aspirin (30). Low adherence to aspirin, different dosing and timing may explain why some studies have not found aspirin to be preventative of LOP. Further reviews and meta-analyses have also found aspirin to be preventative of pre-eclampsia among high-risk patients (55, 56). Yet this data does not focus on LOP specifically. It is necessary to explore these findings for LOP with randomized control trials among high-risk cohorts.

### 4.3 Conclusion and future directions

This review finds that key risk factors for LOP are BMI, chronic hypertension, high MAPs, nulliparity, and maternal age. The strongest predictors for LOP are chronic hypertension and an elevated first-trimester MAP. Chronic hypertension gave the highest odds ratio for LOP and an elevated MAP was the most common significant predictor identified across all studies.

Further studies should aim to use the identified risk factors in combination with other markers to form a clinically useful predictive model for LOP. Biomarkers for LOP need to be found, and perhaps looking additionally in the second or third trimester among large cohorts would yield useful results as this is when the pathogenesis is hypothesized to occur.

The present review demonstrates that aspirin may be a promising preventor for LOP among these women at high-risk. Further preventative measures are needed for patients not deemed to be at high risk, or which can be implemented at a later gestation. It is likely that the biomarkers which will identify patients at high risk of LOP would also aid in finding preventative measures.

## Data Availability

The original contributions presented in the study are included in the article/supplementary material, further inquiries can be directed to the corresponding author.
